# The scent of wolves: pyrazine analogs induce avoidance and vigilance behaviors in prey

**DOI:** 10.3389/fnins.2015.00363

**Published:** 2015-10-07

**Authors:** Kazumi Osada, Sadaharu Miyazono, Makoto Kashiwayanagi

**Affiliations:** ^1^Division of Physiology, Department of Oral Biology, School of Dentistry, Health Sciences University of HokkaidoIshikari-Tobetsu, Japan; ^2^Department of Sensory Physiology, Asahikawa Medical UniversityAsahikawa, Japan

**Keywords:** fear, Hokkaido deer, kairomone, pyrazine analogs, wolf

## Abstract

The common gray wolf (*Canis lupus*) is an apex predator located at the top of the food chain in the Northern Hemisphere. It preys on rodents, rabbits, ungulates, and many other kinds of mammal. However, the behavioral evidence for, and the chemical basis of, the fear-inducing impact of wolf urine on prey are unclear. Recently, the pyrazine analogs 2, 6-dimethylpyrazine, 2, 3, 5-trimethylpyrazine and 3-ethyl-2, 5-dimethyl pyrazine were identified as kairomones in the urine of wolves. When mice were confronted with a mixture of purified pyrazine analogs, vigilance behaviors, including freezing and excitation of neurons at the accessory olfactory bulb, were markedly increased. Additionally, the odor of the pyrazine cocktail effectively suppressed the approach of deer to a feeding area, and for those close to the feeding area elicited fear-related behaviors such as the “tail-flag,” “flight,” and “jump” actions. In this review, we discuss the transfer of chemical information from wolf to prey through the novel kairomones identified in wolf urine and also compare the characteristics of wolf kairomones with other predator-produced kairomones that affect rodents.

## Introduction

The common gray wolf (*Canis lupus*) is an apex predator at the top of the food chain in the Northern Hemisphere. It preys on rodents, rabbits, ungulates, and many other kinds of mammal. The detection of predator phenotypic traits by prey species is a vitally important function of communication between mammals. How prey species discern predators is an important question. For prey animals that rely on chemical communication to regulate social and sexual interactions, it is possible that the presence of a predator can be detected by its scent. These scents and some non-volatile molecules that affect the vomeronasal organ (VNO) (Hurst et al., 2001; Kimoto et al., 2005; Papes et al., 2010; Kaur et al., 2014) are defined as semiochemicals. Semiochemicals are divided into two major groups: pheromones (for conspecific communication) and allelochemicals (for interspecific communication) (Nielsen et al., 2015). Kairomones are allelochemicals that transfer unidirectionally from an emitter to a receiver and provide a benefit to the receiver organism (Brown et al., 1970; Liberles, 2014; Nielsen et al., 2015; Wernecke et al., 2015). Therefore, when a prey animal benefits, the chemical signal produced by a predator is a kairomone. The known kairomones produced by predators that affect rodents are summarized in the Table 1.

**Table 1:** **Rodent kairomones and the source materials from which they were derived**.

**Kairomones**	**Structure**	**Source**	**References**
2-propylthietane, 3-propyl-1,2-dithiolane	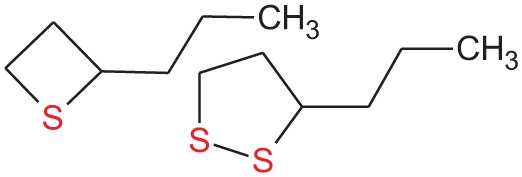	Anal grand secretions from stoats (*Mustela erminea*) and ferrets (*M. putorius*)	Crump, 1978, 1980; Crump and Moors, 1985; Sullivan et al., 1988a,b
Trimethylthiazoline	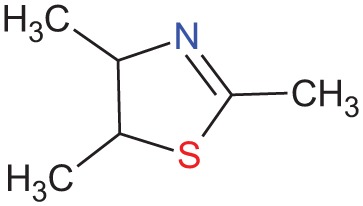	Feces from red foxes (*Vulpes vulpes*)	Vernet-Maury et al., 1984; Wallace and Rosen, 2000; Fendt et al., 2005
MUP-13, MUP Feld4	Major urinary proteins: MW 18,729 kD (MUP-13)	Urine from rats *(Rattus norvegicus*), and saliva from cats (*Felis catus*)	Papes et al., 2010
Phenylethylamine	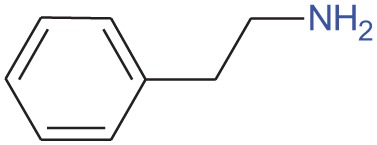	Urine from various kinds of carnivore	Ferrero et al., 2011
Alkylpyrazine analogs	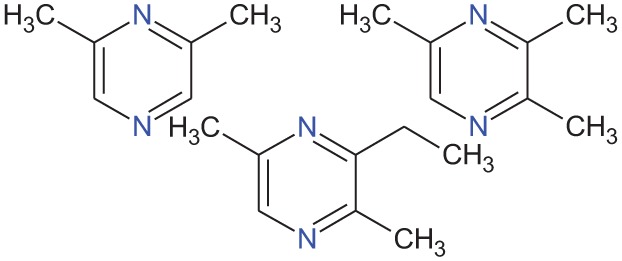	Urine from wolves (*Canis lupus*)	Osada et al., 2013

Wolf urine contains several volatile chemicals that could be used for predator-prey chemosignaling. Wolves use scent-marking to inform neighboring wolf packs of their existence, and herbivores may also use the signals. Moreover, wolf urine is artificially applied by humans to keep many kinds of herbivore and other animals at bay (Sullivan et al., 1985a,b; Pyare and Berger, 2003; Chamaillé-Jammes et al., 2014). Thus, wolf urine possibly includes unidentified molecules that are used in predator-prey chemosignaling. Actually, the behavioral evidence for, and the chemical basis of, the fear-inducing impact of wolf urine on prey species were recently unveiled (Osada et al., 2013).

Wild animals frequently invade human habitats and can cause serious problems. For example, deer cause large amounts of damage and economic losses in agricultural, horticultural, and forest resources around the world (Trdan and Vidrih, 2008; Killian et al., 2009; Kimball et al., 2009; Baasch et al., 2010; Gheysen et al., 2011; Masuko et al., 2011). Rather than hunting deer, it may be preferable to control their behavior using kairomones so that they can coexist with other wild animals without destroying human habitats or natural environments. Here, we discuss the transfer of chemical information from wolf to prey through novel kairomones identified in wolf urine and compare the characteristics of the wolf kairomones with those of the other predator-produced kairomones that affect rodents.

## The study of chemical communication via wolf urine

### The scent of wolf urine is used for social communication

Because wolves are gregarious carnivores, their olfactory-based communication system is much more complex than that of comparatively solitary species such as foxes and cats (Fox and Cohen, 1977). In addition to olfactory cues, wolves use visual, vocal, and tactile cues to communicate with each other. However, urine is an important mode of chemical communication for the wolf (Peters and Mech, 1975; Mech, 1977).

Using radio-tracking techniques to study natural wolf packs, Peters and Mech (1975) identified detailed conditions for urine scent-marking in wolves. Wolf packs living in the Superior National Forest of northeastern Minnesota are territorial, and most stable territories range in size from 125 to 310 km^2^. These territories seem to be stable and exclusive, and over several months there is a “buffer zone” (about 2 km wide) along the borders. Interpack conflict is rare or non-existent in the area.

During four winter seasons, scent marking was studied to clarify the role played by marking in the information flow that is integral to maintaining the organization of wolf populations. One of the most important spatial differences was the difference in the urination rate between the centers and the edges of wolf territories. The frequency of urination (number of urinations/km track) along the edges of territories was approximately 2.1-fold higher than that in the center of territories. The wolves engaged in scent marking along the edges of their territories to inform members of the neighboring wolf packs of their presence, particularly during the breeding season (Peters and Mech, 1975). Similarly, coyotes (*Canis latrans*), which are smaller close relatives of the wolves, scent-mark in the same manner to maintain their territories (Gese and Ruff, 1997). These observations suggest that alarm pheromones used by conspecifics exist in wolf urine.

It is also conceivable that wolf urine contains chemosignals used for communication between individuals belonging to different species. For example, densities of white-tailed deer (*Odocoileus virginianus*) are higher in buffer zones between territories held by wolf packs than inside the territories (Mech, 1977). Wolves apparently avoid hunting in buffer zones between the edges of territories that are indicated by scent-marks. Therefore, the survival of deer should be greater in buffer zones due to lower rates of predation by wolves. Importantly, deer voluntarily migrate to buffer zones in winter (Hoskinson and Mech, 1976; Rogers et al., 1980), suggesting that deer utilize chemo-olfactory cues and other sensory cues to reach these safety zones. Deer must be able to assess the quality and the intensity of odor emitted from the urine of wolves. Research therefore clearly suggests that urination by wolves and the semiochemicals in the urine are involved not only in conspecific pheromone perception, but also in the interspecific detection of kairomones.

### The urine of predators, including wolves, causes avoidance behavior in various types of herbivore

Research shows that exposure to predator odor induces avoidance behavior in many kinds of prey animal, including ungulates. For example, white-tailed deer and/or black-tailed deer (*Odocoileus hemionus columbianus*) avoid the urinary odor of predators, including wolf, coyote, red fox (*Vulpes vulpes*), wolverine (*Gulo gulo*), lynx (*Lynx canadensis*), and bobcat (*Lynx rufus*), and the odor of the feces of cougar (*Puma concolor*), coyote, and wolf (Sullivan et al., 1985b; Swihart et al., 1991).

Specifically, Swihart et al. (1991) demonstrated that the topical application of coyote and bobcat urine (6 ml per plant) at weekly intervals to Japanese yew (*Taxus cuspidate*) and eastern hemlock (*Tsuga canadensis*) trees deterred white-tailed deer for at least 8 weeks. Non-predator (human and rabbit *Sylvilagus floridanus*) urine had no repellent effect. Coyote urine prevented damage slightly less well than bobcat urine. On the other hand, Sullivan et al. (1985b) conducted a bioassay to study the effect of dispensed predator urines in vials attached to salal (*Gaultheria shallon*) branches on black-tailed deer. The study demonstrated that the odor of wolf, coyote, and fox urine was more effective in suppressing the feeding of deer on salal than control or bobcat urine; this effect lasted for at least 6 days. Moreover, cattle (*Bos taurus*) exposed to the odor of wolf or dingo (*Canis lupus dingo*) showed defensive or avoidance responses (Kluever et al., 2009). These studies suggest that predator urine contains kairomone(s), which induce robust avoidance behavior not only in wild deer, but also in ungulate livestock.

In addition, hares (*Lepus americanus*) leave or avoid areas treated with odors derived from several kinds of predator (Sullivan et al., 1985a). American beavers (*Castor canadensis*) and marsupials show defensive or avoidance responses to the odor of wolves or dingoes (Lindgren et al., 1995; Parsons and Blumstein, 2010). Importantly, exposure to the urine of predators, but not to that of herbivores or conspecifics, induces defensive behaviors in laboratory rats (*Rattus norvegicus*), suggesting that laboratory rats detect a predisposed-active cue in predator odors (Fendt, 2006).

Research clearly indicates that the urine and feces of many carnivores, including wolves, contains kairomones that repel their prey animals. In a practical application, the urine of wolves or other predators can be used to drive away these animals without killing them (Sullivan et al., 1985a,b; Lindgren et al., 1995; Severud et al., 2011).

### Chemical physiology of the volatile constituents in wolf urine and that of other wild canids

The urine of the wolf (Raymer et al., 1984), coyote (Nolte et al., 1994) and red fox (Jorgenson et al., 1978) contains numerous chemicals that emit a strong stench. Raymer et al. (1984) analyzed the chemical components of wolf urine that change with gender. Gas chromatography-mass spectrometry (GC-MS) and gas chromatography with a flame ionization detector (FID-GC) were used to identify and quantify typical wolf urinary components. The profile of volatiles resulting from GC separation was obtained through a headspace sampling procedure by thermal desorption of volatiles from a porous polymer (Tenax). Several compounds including Δ^3^-isopentenyl methyl sulfide (IMS), 3, 5-dimethyl-2-octanone, and acetophenone were clearly associated with the gender of the animal, and also changed seasonally (Raymer et al., 1984). Therefore, it is postulated that the production of these wolf urinary chemicals depends on reproductive hormones. In castrated male wolves, testosterone induces the formation of some compounds typically associated with the intact male (several types of middle chain alkyl ketones and alkyl sulfides), while reducing the levels of other compounds (i.e., 3-ethylcyclo-pentanone and acetophenone) associated with castrated males and females (Raymer et al., 1986). Similarly, four volatile chemicals (IMS, 2-phenylethyl methyl sulfide, 6-methyl-heptene-2-one, and geranylacetone) were identified as constituents of the urine of red foxes (both sexes), with greater production during the winter season when mating occurred (Jorgenson et al., 1978).

In addition to the reproductive hormones and the season, the diet of a predator (the coyote) affected the ability of the urine to cause avoidance behavior in prey (Nolte et al., 1994). The authors used four species of rodent, namely the mountain beaver (*Aplodontia rufa*), the house mouse (*Mus musculus*), the deer mouse (*Peromyscus maniculatus*), and the guinea pig (*Cavia porcellus*) as subjects in behavioral experiments. Urine samples were collected from four urine donor coyotes that each ate only cantaloupe melon (*Cucumis melo*) (FU) or only minced raw meat (MU) for 5 days while housed in metabolic chambers. After acclimatization, the four species of rodent were given 24 h two-choice tests between apple cubes associated with either the FU or the MU. In this choice test, all four types of rodent ingested significantly more apple cubes from bowls scented with FU than they did from bowls that contained MU. Thus, all four species of rodent avoided the MU odor in favor of the FU odor. The results from high-performance liquid chromatography analysis of the urine showed that two unidentified chemical peaks existed only in MU. When MU was treated with mercuric chloride, these two peaks disappeared and the avoidance behavior evoked by the MU odor also decreased (Nolte et al., 1994). These data suggest that sulfurous metabolites of meat digestion are important for the repellent nature of predator odors for potential prey. Additionally, although these authors found that the FU and the sulfur-deprived MU (SR) were both less aversive to prey than the MU, the intake of food was reduced in the presence of FU (and SR) relative to a control. This avoidance, therefore, might be attributed to other non-sulfurous compounds (Nolte et al., 1994).

When combined, it is conceivable that IMS (Wilson et al., 1978) and its derivatives are the candidates of predator urinary kairomones of wild canids, including the wolf. However, the capacity of these synthesized chemicals to induce vigilance behaviors in prey is limited, at least in a field experimental setting. For example, Sullivan et al. (1988a) demonstrated that when IMS or the analog (3-methyl-3-butenyl methyl sulfide; MBMS) was dispensed within capillary tubes and attached to apple trees with a twist-tie, there was no significant reduction in feeding damage to these trees from meadow voles (*Microtus pennsylvanicus*). Similarly, IMS and its analog did not cause significant avoidance and/or vigilance behaviors by pocket gophers (*Thomomys talpoides*) (Sullivan et al., 1988b), Mountain beavers (*Aplodontia rufa*) (Epple et al., 1995), or ungulates (Lindgren et al., 1995; Nolte et al., 2001). Only the snowshoe hare (*Lepus americanus*) effectively avoided the MBMS (Sullivan and Crump, 1986). Therefore, wolf urine likely contained additional kairomones that were used in predator-prey chemosignaling.

## Identification of wolf kairomones by mice

### Alkyl pyrazine analogs are wolf kairomones

**Figure 1:**
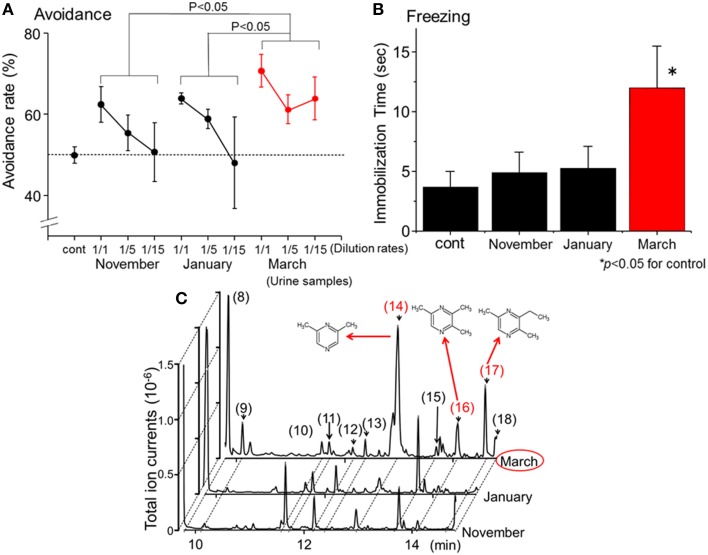
**Identification of novel kairomones (pyrazine analogs) in wolf urine. (A)** Avoidance rates observed during exposure of mice to wolf urine samples harvested in approximately November 2009, January 2010, and March 2010. The avoidance rate was defined as the amount of time spent in the short arm of a Y maze in the presence of the control odor (water), divided by the total amount of time spent in both short arms in the presence of the wolf urine odor or the control odor. The statistical significance of the differences between the avoidance rates elicited by each of the wolf urine samples was assessed by repeated-measures ANOVA followed by Fisher's PLSD *post-hoc* test. **(B)** Comparison of the duration of “freezing” (immobilization behavior) by mice during a 3 min exposure to five-fold diluted wolf urine samples. The statistical significance of the differences between the freezing duration in response to wolf urine samples was compared with control (water) by means of ANOVA followed by Dunnett's *post-hoc* test. **(C)** Chromatograms from GC-MS analyses of wolf urine samples. Numbers refer to the following compounds: (8) Δ^3^-isopentenyl methyl sulfide; (9) 1-(methylthio)-2-methylbut-2-ene; (10) 3-buten-1-ol, 3-methyl-; (11) 4-methyl-3-heptanone; (12) 2,4-dithiapentane^*^; (13) 1-pentanol, 2-methyl-; (14) pyrazine, 2,6-dimethyl- (DMP)^*^; (15) dimethyl trisulfide^*^; (16) pyrazine, trimethyl- (TMP)^*^; (17) pyrazine, 3-ethyl-2,5-dimethyl (EDMP)^*^; (18) acetic acid^*^. ^*^Identified by GC-MS (*n* = 6) and by comparison with the retention times of identified chemicals. All figures modified from Osada et al. (2013).

In a previous study (Osada et al., 2013), the avoidance (Fendt, 2006; Ferrero et al., 2011) and freezing behaviors (Wallace and Rosen, 2000; Fendt et al., 2005; Buron et al., 2007; Fendt and Endres, 2008) of female house mice in response to wolf urine were systematically analyzed. Three sets of commercially available urine samples, which were harvested approximately in November 2009, January 2010, and March 2010, from both genders of wolves that belonged to the same pack (*n* > 10) were obtained. As mentioned in the previous sections, the odor and the chemical components contained in wolf urine depend on the diet, the season, and the hormonal status of the animal. Therefore, differences might be expected in the avoidance behavior induced in mice by these different urine samples. However, all undiluted samples induced significant avoidance behavior in the mice when compared with the control (Figure 1A).

Predator scents comprise complex cocktails of volatiles, some of which emit a strong stench. Endres and Fendt (2009) showed that trimethylthiazoline (TMT), a kairomone derived from fox feces, induces freezing behavior in prey at very low concentrations. However, butyric acid, which is a repugnant, non-predator odor, did not induce such behaviors. Therefore, to determine which of the urine samples contained the most kairomones, avoidance, and freezing behavior bioassays were conducted using diluted urine samples. Osada et al. (2013) found that one group of urine samples, those harvested in March, induced the strongest vigilance behavior in mice (Figures 1A,B); these results indicate that the levels of kairomones in wolf urine might also increase near the end of the breeding season.

To identify potential novel kairomones in wolf urine, GC-quadrupole MS in conjunction with headspace solid phase micro-extraction was conducted. From over 50 representative peaks, 2, 6-dimethylpyrazine (DMP), 2, 3, 5,-trimethylpyrazine (TMP), and 3-ethyl-2, 5-dimethyl pyrazine (EDMP) were among several volatiles present at higher concentrations in the urine sample collected in March than in samples collected at other times (Figure 1C; peaks (14), (16), and (17), respectively). The concentration of 1-(methylthio)-2-methylbut-2-ene (peak number 9) tended to be highest in the March urine samples, although the difference was not statistically significant. Although these volatiles were characterized by a strong odor, there were no previous reports suggesting that they facilitate conspecific communication among canines (Jorgenson et al., 1978; Raymer et al., 1984). Therefore, these volatiles were hypothesized to be novel kairomones in the urine of wolves (Osada et al., 2013).

The results of additional behavioral and immunohistochemical studies indicate that these pyrazine analogs, especially a cocktail thereof, elicit significant freezing behavior in mice, at least in part by stimulating the murine accessory olfactory bulb (AOB). Thus, the pyrazine analogs identified in wolf urine represent a set of novel kairomones that initiate fear-related behavior in mice.

### The putative sensory system involved in inducing freezing and avoidance behavior in response to kairomones for rodents

For several reasons, it is likely that these pyrazine analogs stimulated the main olfactory epithelia (MOE) to induce the freezing behavior. First, most of the alkyl pyrazine analogs are volatile compounds that emit a pungent odor (Tsantili-Kakoulidou and Kier, 1992). Second, measured as the uptake of [^14^C] 2-deoxyglucose, Johnson et al. (2005) demonstrated that 2,3-dimethylpyrazine caused a robust stimulation of the glomerular layer of the rat main olfactory bulb (MOB). Third, the freezing behaviors are only observed in response to repugnant predator odors, such as TMT (Papes et al., 2010). In addition to the pyrazine analogs, there are several other predator odorants that elicit significant vigilance behaviors in rodents (Table 1). For example, Vernet-Maury (1980) reported that TMT is the primary component of the odor of fox feces, and that it induces autonomic and behavioral anti-predator responses in rodents. For example, experimental rats and mice exposed to the odor of foxes or to TMT (Vernet-Maury et al., 1984; Fendt et al., 2005) showed fear-related response behaviors, such as freezing-in-place (Wallace and Rosen, 2000; Buron et al., 2007; Fendt and Endres, 2008; Janitzky et al., 2009). Kobayakawa et al. (2007) demonstrated that TMT is mainly detected in the dorsal domain of the MOB. Similarly, rodents exposed to cat-derived odors displayed dose-dependent vigilance responses, including freezing, avoidance, and the increased production of stress hormones (Takahashi et al., 2005, 2007, 2008). Although little is known about the chemical basis underlying cat odor-induced freezing behavior, volatile compounds containing 3-mercapto-3-methyl-1-butanol have been identified as species-specific odorants in cat urine. These sex- and age-dependent cat-specific volatile compounds (Miyazaki et al., 2006) are detected as territorial markers and are used in conspecific recognition or in female attraction by mature male cats (Miyazaki et al., 2008). To detect predator signals, rodents may also use these volatiles. Using a reporter gene assay with trace amine-associated receptors (TAARs), Ferrero et al. (2011) found that the mouse TAAR4 selectively responded to the urine of several carnivores. Then, bobcat urine was fractionated with silica gel chromatography and analyzed with the reporter gene assay. The results showed that 2-phenylethylamine, a common component of the urine of various carnivores was a key component of an odorant blend that triggers spontaneous aversion via the olfactory sensory neurons (Table 1). In contrast to the TMT and the predator-derived lipocalins (Papes et al., 2010; see below), 2-phenylethylamine was identified in the urine of many species of carnivore and therefore might enable prey to avoid novel and dangerous predators (Liberles, 2014). Taken together, most of the above-mentioned volatile chemicals act by stimulating the MOE.

In addition to the primary olfactory system, most mammals have a vomeronasal system; this system contributes to the detection of certain conspecific pheromones, and it also perceives common volatile odorants (Trinh and Storm, 2003; Brennan and Keverne, 2004). Moreover, the vomeronasal system is thought to detect interspecific kairomones. For example, Ben-Shaul et al. (2010) identified a significant set of murine AOB neurons that respond robustly and selectively to predator cues. In addition, the exposure of rodents to cat odors increased the number of Fos-positive cells in the AOB (Staples et al., 2008). However, the chemical composition of these kairomones in predators remains difficult to determine. Papes et al. (2010) showed that in VNO-defective animals, TrpC2^−∕−^, the odor from mice predators (urine from rats, neck swab of cats, shed skin of snakes) did induce avoidance and risk assessment behaviors. The authors then purified the kairomones using size extraction fractionation and anion exchange FPLC techniques and identified the kairomones using behavioral and Ca^2+^ imaging assays. They demonstrated that the major urinary protein of rat (lipocalin) and recombinant feline Mup (based on Mup Feld4 in cat saliva) (Table 1) are sufficient to activate VNO and AOB neurons and initiate both defensive behavior and the ACTH response.

Osada et al. (2013) suggested that pyrazine analogs stimulated the AOB; therefore, they examined the immunoreactivity of Fos, a marker of neuronal excitation, primarily in the AOB. They found that the immunoreactivity of Fos was different in the AOB, particularly in the posterior granule cell layer, after mice were exposed to fresh wolf urine samples. These results suggest that substances in wolf urine cause excitation in part of the vomeronasal system. In this regard, pyrazine analogs might be the first identified volatile urinary chemosignals that evoke fear-associated immobilization and stimulate the rodent AOB and perhaps the MOB as well. Further studies are in progress to clarify the precise neurophysiological mechanisms underlying hard-wired fear-related responses evoked by pyrazine analogs.

In addition to the MOE and the VNO, the Gruenberg Ganglion is a detector of kairomones. According to previous studies, principal anal gland compounds from the stoat (*Mustela erminea*) and ferret (*M. putorius*) markedly alter the distribution of gophers (*Thomomys talpoides*) and clearly reduce the feeding of meadow voles on apple trees (Crump, 1978, 1980; Crump and Moors, 1985; Sullivan et al., 1988a,b). The predator odor chemicals involved comprise alkylthietanes and dithiolane, which were ether-extracted from excretions of the anal gland. Additionally, Brechbühl et al. (2013) demonstrated that 2-propylthietane, TMT, and 2-sec-butyl-4, 5-dihydrothiazole elicit freezing behavior in C57BL/6J mice by stimulating the Gruenberg Ganglion. Interestingly, several alkyl pyrazines, including DMP and TMP, can induce Fos-positive Gruenberg Ganglion cells in a dose-dependent manner (Mamasuew et al., 2011). Therefore, it is conceivable that these pyrazine analogs also induce robust fear-related behaviors by stimulating the Gruenberg Ganglion.

### Putative mechanism for the production of pyrazine analogs in predator urine

The mechanism(s) by which pyrazine analogs are produced in wolf urine is unknown. However, an intriguing possibility is related to glycation, which occurs in all living animals (McPherson et al., 1988; Fu et al., 1992). Alkylpyrazine analogs are a typical class of glycation compound (Adams et al., 2008), which are formed between reducing sugars and glycine oligopeptides (Lu et al., 2005). Actually, food-derived oligopeptides can be detected in the blood after oral ingestion of meat and collagen (Iwai et al., 2005; Bauchart et al., 2007). Therefore, it is conceivable that the blood glucose and amino compounds derived from foods containing meat or connective tissue may be the source of pyrazine analogs generated in the urine of wolves and, perhaps, other carnivores.

## The effect of putative kairomones in wolf urine on ungulates

### Aversion and vigilance behaviors in hokkaido deer exposed to pyrazine analogs—a field experiment

**Figure 2:**
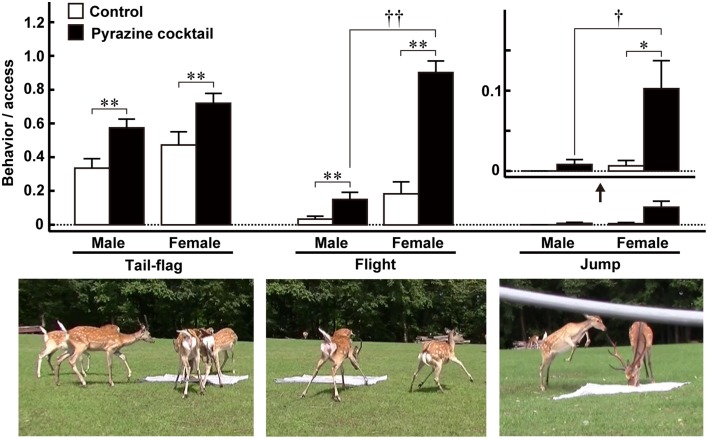
**Fear-related behaviors of male and female Hokkaido deer evoked by pyrazine analogs in the feeding experiment**. The proportion of tail-flag, flight, and jump actions were estimated by calculating the ratio of the number of times each action was performed by males (*n* = 27) and females (*n* = 19) to the number of deer accessing the feeding areas. Open and closed bars indicate control and pyrazine cocktail feeding areas, respectively. ^*^p < 0.05, ^**^p < 0.01, Wilcoxon signed-rank test. ^†^*p* < 0.05, ^††^*p* < 0.01, Mann-Whitney U-test. Lower panels show typical photographs of each of the fear-related behaviors. Modified from Osada et al. (2014).

As mentioned above, Osada et al. (2013) identified a set of pyrazine analogs as wolf urinary kairomones that induce aversive and freezing behaviors in mice. A cocktail of these compounds had a greater effect than any one component alone. Because the wolf preys on various kinds of mammal, including ungulates, the authors thought that the pyrazine analogs might be kairomones that induce vigilance and fear in large mammals. Therefore, to investigate the ability of the pyrazine cocktail to act as a kairomone in prey animals other than mice, Osada et al. (2014) performed a field experiment on Hokkaido deer (*Cervus nippon yesoensis*). The experiment was conducted in a seminatural deer park (44°12′ N and 142°48′ E, Nishiokoppe, Hokkaido, Japan). Approximately 30 deer inhabited an enclosed area and had free access to herbage, bamboo grass, tree leaves, bark, and water. The feeding experiments were conducted twice, from summer to autumn, 2013. When individual male and female deer were followed, the pyrazine cocktail suppressed the duration and frequency of access to the feeding area by half. The cocktail also led to deer taking twice as long to reach the feeding area. Moreover, the cocktail elicited vigilance behaviors around the feeding areas, such as tail-flag (deer lift up their tail), flight (deer rapidly escape with their necks retracted), and jump (deer spring back; Figure 2). These behaviors might indicate fear and may act as alarm signals to warn conspecifics of impending danger (Caro, 1995, 2005; Eilam, 2005; Stankowich and Coss, 2006); thus, the results from this field study suggest that the pyrazine cocktail acted as a wolf kairomone, eliciting fear-associated aversive behaviors in deer (Osada et al., 2014).

### Hokkaido deer have “remembered” the scent of a predator, the wolf, for 100 years

The observation that the pyrazine cocktail acted as a wolf kairomone leads to the following question: why do Hokkaido deer show fear-associated aversive responses to the pyrazine analogs that form part of the scent of wolf urine? Japanese wolves (Hokkaido wolf, *C. l. hattai*; Honshu wolf, *C. l. hodophilax*) have been extinct for the last 100 years (Walker, 2005), so the individual deer used in the experiment have never been under threat of predation by wolves. Thus, the pyrazine cocktail may elicit a predisposed fear response in deer. Numerous studies demonstrate the persistence of responses to the scent of predators, and to their kairomones, in laboratory strains of rodents that have not experienced predation for several hundred generations (Apfelbach et al., 2005; Fendt, 2006; Osada et al., 2013; Takahashi, 2014).

Berger (1999) and Pyare and Berger (2003) reported that female moose (*Alces alces*) from a region that overlapped with the territory of a wolf pack (mainland Alaska) showed significantly stronger vigilance behavior when exposed to the odors of wolves than female moose from a region in which wolves were absent for at least 60 years until the 1990s (Wyoming). However, notably, the vigilance behavior was not higher than that of moose in a predator-free region (Kenai Peninsula) suggesting that learning was not a necessary component of the avoidance behavior induced by wolf urine. Additionally, Chamaillé-Jammes et al. (2014) obtained a similar result in a field study conducted on the Haida Gwaii archipelago in Canada, where black-tailed deer exhibit a fear response to wolf urine, even after more than 100 years of wolf absence. Interestingly, the response to wolf urine is greater than the response to urine derived from the black bear (*Ursus americanus*), which is currently present but is potentially a less dangerous predator for black-tailed deer. Moreover, wolves are more efficient predators than black bears, which typically attack fawns and rarely attack adults, and have little success when they do (Zager and Beecham, 2006). The authors concluded that the result is in accordance with “the hypothesis of the innate threat-sensitive foraging,” which states that fear responses of prey species to predator cues should be adjusted to the level of danger posed by the predator. Additionally, based on “the multipredator hypothesis” proposed by Blumstein and colleagues (Blumstein, 2006; Blumstein et al., 2006, 2008), the authors speculated that the response to a more dangerous predator (the wolf) might be maintained by encountering a less dangerous predator if both predators' cues are similar, or if the responses are genetically linked (Chamaillé-Jammes et al., 2014). Therefore, Hokkaido deer may have maintained their recognition of, and response to, wolf scent by having contact with current existing predators such as the Hokkaido brown bear (*Ursus arctos yesoensis*) and the red fox (*Vulpes vulpes schrencki*).

Pyrazine analogs are not only found in the urine of predators but also in a wide variety of plants (Bohman et al., 2012, 2014), insects (Tentschert et al., 2000; Sharma et al., 2011), terrestrial vertebrates (Novotny et al., 1986; Woolfson and Rothschild, 1990; Zhang et al., 2005) and foods (EFSA Panel on Food Contact Materials Enzymes Flavourings and Processing Aids (CEF), 2011). This widespread distribution suggests that pyrazine analogs are of special significance as semiochemicals for different types of organism. However, we cannot preclude the possibility that these deer were previously exposed to other substrates that contain identical or similar components and, therefore, might display a learned fear response to the pyrazine mixture. Further research is required to clarify this point.

### Sexual dimorphism in vigilance behaviors in response to wolf scent

Of the fear-related vigilance behaviors of deer quantified in the field experiment, flight and jump behaviors occurred frequently in females in the presence of the pyrazine cocktail (Figure 2; Osada et al., 2014). Although more data are needed before we can draw a firm conclusion, we also believe that the male deer with the largest antlers were less likely to show vigilance behaviors in response to the pyrazine cocktail than the other males and females. Thus, the response of deer to the pyrazine cocktail representing wolf scent is partly dependent on their sex, and, possibly, on the position of males in the social hierarchy of the herd.

Sexual dimorphism in the fear response has been studied in laboratory animals. For instance, in laboratory rats, mice, and meadow voles, females exhibit stronger responses to the odor of a predator (the red fox) than males (Perrot-Sinal et al., 1996; Hubbard et al., 2004; Buron et al., 2007). Age-related and hormonal variation has been also reported in the response to predator odors (Hubbard et al., 2004; King et al., 2005). At present, genetic and pharmacological studies have begun to be implemented to investigate the neural circuits that regulate fear of predator odors. For example, Choi et al. (2005) identified LIM (Lin-11, Is1-1, Mec-3) homeodomain transcription factors as molecular markers for the sub-nuclei of the medial amygdala, which is responsive to predator odors. Moriceau et al. (2004) indicated that corticosterone might control the fear response of infant rats to conspecific predator odor, and that the response might be mediated by activation of the basolateral/lateral amygdala. Do Monte et al. (2008) suggested that noradrenergic transmission may modulate the expression of the fear response of rats to cat odor through the dorsal pre-mammillary nucleus. This knowledge is likely to facilitate further studies on sexual dimorphism in fear of predator odor in laboratory animals, and also in wild animals.

### Potential for the novel wolf kairomones to act as repellents for ungulates

Researchers have identified various kairomones derived from odors of predators of rodents (Table 1). To the best of our knowledge, the pyrazine cocktail is the first example of kairomones that elicit aversive behavior in both rodents and ungulates. Excessively large deer populations may cause economic losses in agricultural, horticultural, and forest resources. To minimize such losses, natural odor sources are frequently used as chemical repellents for ungulates (Apfelbach et al., 2005; Kimball et al., 2009). Synthetic odors, however, have had little or no effect so far (Apfelbach et al., 2005). Because of its aversive effect on deer, the pyrazine cocktail might be effective as a chemical repellent for deer. Clearly, an excellent repellent must have a persistent effect. Studies confirm that the pyrazine cocktail had a good repellent effect when tested on the same herd of deer, and the effect lasted at least 1 month after the first day of the experiment (Osada et al., 2014).

An ideal repellent should also be a natural product. The pyrazine analogs in the cocktail are natural, non-carcinogenic, and of low acute toxicity; indeed, they are responsible for the characteristic roasted aromas in foods such as coffee, peanuts, beef, and potato (EFSA Panel on Food Contact Materials Enzymes Flavourings and Processing Aids (CEF), 2011). Actually, some kinds of alkyl pyrazine are widely used as flavoring ingredients in foods (Burdock and Carabin, 2008). Therefore, the pyrazine cocktail is expected to be an effective deer repellent that will not damage the natural environment.

## Conclusions

We first reviewed historical studies relating to chemical communication between wolves. Urinary chemical communication between conspecifics in wolf packs is important in wild habitats. Moreover, the urine of wolves is also used as kairomones by prey animals. Next, we discussed the identification, chemical basis, and putative sensory system of wolf kairomones compared with other kairomones that affect rodents. We presented the possibility that wolf urine, and the pyrazine analogs contained therein, provoke a fear response by stimulating three different sensory systems, namely, the murine vomeronasal system, the main olfactory system and, perhaps, the Gruenberg Ganglion. In future, a number of novel semiochemicals which stimulate the three sensory systems may be found. We then discussed studies showing that these pyrazine analogs elicited vigilance behaviors not only in rodents, but also in an ungulate, the Hokkaido deer. In this section we discussed how Hokkaido deer have remembered the scent of wolves for 100 years or more. In addition, the sexual dimorphism in vigilance behavior and the potential of wolf kairomones to act as repellents were mentioned. Further studies are required to determine whether the vigilance response of deer to the pyrazine cocktail is predisposed or learned. Moreover, the discovery that pyrazine analogs evoke vigilance behaviors in prey animals provides a strong rationale for additional studies of odorant-induced behaviors and the neurophysiological mechanisms underlying them.

### Conflict of interest statement

The authors declare that the research was conducted in the absence of any commercial or financial relationships that could be construed as a potential conflict of interest.
